# Energy Neutral Wireless Bolt for Safety Critical Fastening

**DOI:** 10.3390/s17102211

**Published:** 2017-09-26

**Authors:** Biruk B. Seyoum, Maurizio Rossi, Davide Brunelli

**Affiliations:** Department of Industrial Engineering, University of Trento, Via Sommarive, 9, 38123 Trento, Italy

**Keywords:** smart bolt, TEG, Peltier, IoT, energy harvesting

## Abstract

Thermoelectric generators (TEGs) are now capable of powering the abundant low power electronics from very small (just a few degrees Celsius) temperature gradients. This factor along with the continuously lowering cost and size of TEGs, has contributed to the growing number of miniaturized battery-free sensor modules powered by TEGs. In this article, we present the design of an ambient-powered wireless bolt for high-end electro-mechanical systems. The bolt is equipped with a temperature sensor and a low power RF chip powered from a TEG. A DC-DC converter interfacing the TEG with the RF chip is used to step-up the low TEG voltage. The work includes the characterizations of different TEGs and DC-DC converters to determine the optimal design based on the amount of power that can be generated from a TEG under different loads and at temperature gradients typical of industrial environments. A prototype system was implemented and the power consumption of this system under different conditions was also measured. Results demonstrate that the power generated by the TEG at very low temperature gradients is sufficient to guarantee continuous wireless monitoring of the critical fasteners in critical systems such as avionics, motorsport and aerospace.

## 1. Introduction

The average number of sensors on electromechanical systems (EMS), which includes different aerial and ground vehicles, complex infrastructures such as manufacturing plants, underwater oil & gas pipelines has undergone an exponential increase. Those EMS building blocks that are not fitted with sensors and therefore are not monitored and controlled by an automation system in real-time must undergo a periodic manual inspection and maintenance. This manual inspection which has a probability of being either premature or overdue is unreliable and adds extra costs. The effort of guaranteeing the safety by continuously monitoring the parts of EMS is the reason behind the expansion in the number of on-system sensors.

In EMS, critical fasteners that are located close to a large source of heat, such as an engine, are subject to a continuous rapid heating and cooling which will eventually render them brittle [1]. The fasteners (bolts) are also subject to wear and tear caused by being over stressed. Over time and due to movement, bolts may also become loose and unable to provide the proper tension to hold the parts together. It is therefore important to monitor the health of critical fasteners in EMS, especially those whose failure can lead to a total failure or significant reduction in performance of the whole EMS.

The safety and average time before the need for a replacement of critical fasteners can be determined using a continuously recorded temperature data. The tension in the fasteners can also be monitored to have feedback about the loosening of the bolts. Bolts that are crucial for the proper functioning of the whole system usually have a large diameter which makes them ideal to fit small temperature sensors with an intelligent circuitry within the head.

**Figure 1:**
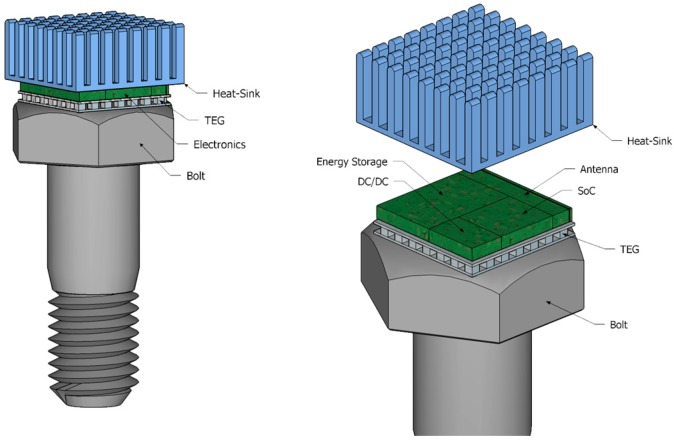
System Model.

The purpose of this work is to develop a system that monitors the safety of critical fasteners. The system has a small form factor to fit on the head of the bolt with the embedded temperature sensors. The heart of the system is a low power micro-controller with an integrated radio module to send the sensed data to a central controller. The module will be powered from a TEG that generates electricity from the temperature gradient between the surface of the critical fastener and the environment. Figure 1 is a schematic representation of the architecture of the envisioned system. In terms of performance the system should fulfill the following requirements

1. Generate its own energy
2. Monitor available energy before transmission and postpone transmission if available energy is below a threshold
3. Dynamically adjust its duty cycle (CPU wakeup frequency) based on available energy from TEG
4. Dynamically adjust radio transmission power (above a certain threshold) based on available energy and environmental conditions
5. Stay alive for as long as possible

The remainder of this paper is organized as follows. Section 2 provides a brief survey of the state of the art in terms of TEG powered applications. In Section 3, the experimental setup is explained, and in Section 4 the obtained results are presented with the discussion. Finally, a conclusion is drawn based on the results.

## 2. Related Work

Monitoring critical fasteners is an issue that several companies are recently trying to address. For example, Stress Indicators Incorporated [2] is a company that produces smart bolts with a built-in visual tension indicator. This product measures tension on the bolt and displays the result via a shade of red LED mounted on the top of the bolt. Although this is an innovative product, it is not suitable for a coordinated sensing as it would be difficult to interface it into an existing closed loop control system. In [3], General Motors employed smart bolts with a combination of small memory and RFID heads. These bolts are mounted on the engine blocks. As the engine passes through the assembly line, data is read and written to the RFID tag by the machines. This data is used at subsequent stages to verify if correct operations were done at the previous stage of assembly. Here the concern was so much into storing state information during the engine assembly rather than monitoring the health of the bolt itself. To the best of our knowledge, even if wireless sensor networks is a consolidated technology for more than ten years (e.g., [4]), we have not come across any other publication or product that monitors different properties of bolts in real time and relays gathered data to a central controller, while many works are targeting TEGs to build a reliable power supply for wireless sensor in industrial environments [5,6,7]. An interesting solution in this field is to exploit Phase-Change Materials (PCMs) to maximize the energy extracted from a thermal gradient, specifically in those cases where temperature gradients are not constant and reverse thermal gradient may cyclically occur [8], for example in airplanes during take-off and landing phases [9].

In the field of micro-fabrication process, different groups work towards the definition of a reliable, easy to industrialize [10] and possibly complementary metal-oxide semiconductor (CMOS) compatible [11] fabrication process to enable a fast and seamless integration of thermoelectric power supply within the very same integrated electronics that realize the monitoring task [12]. For example, authors in [13] developed and characterized soil moisture sensor within the same ceramic substrate housing a custom thermoelectric generator.

In terms of characterization of TEGs to find the maximum power, Gao et al. [14] proposed an additional numerical analysis to improve the estimation of maximum power by measuring the open circuit voltage and short circuit current, in addition to the traditional approach as presented for example in [15]. A non-invasive thermal resistance estimation of TEGs is also presented in [16,17]. In a different approach [18], presents a model to predict the harvested energy using TEGs and photovoltaics based on parameters such as light intensity, temperature gradient, human activity. This model can be adapted to perform theoretical analysis prior to performing any experiment on TEGs.

A common application of TEGs is harvesting small energy to power electronics with low power consumption. Since the very early attempts of using TEGs without intermediate power conditioning for ultra-low power applications (as in [19,20]), there have been a lot of progresses in harnessing thermoelectricity for different applications. The possibility of powering a network of wireless sensors using a combination of TEGs and DC-DC boosters is explored in [21]. In this work, the DC-DC booster efficiency was roughly approximated between 10–50% and the sensor module with an on-board ZigBee radio was operating with a 10% duty cycle. In [22,23], an interesting application of powering gas sensors in a server farm using heat generated from the servers in the data center is proposed. The experiments include the analysis of performance of a combination of two TEGs as generators and two server boards running benchmark applications as the source of thermal energy. The system was functioning with a duty cycle of 0.0027%. Interestingly, authors of [24], demonstrate that in indoor conditions, TEGs exploiting human body warmth can provide a power density (20 µW/cm^2^) larger than that of photovoltaic panels. Similarly, an application on [25] demonstrates the use of TEGs to power sensors used in body area networks for assisting healthy aging. TEGs were powered from body heat scavenging up to 520 µW at 15 °C thermal gradient resulting into a 0.4% duty cycle of the application. In [26], TEGs are used as a power source to monitor deep sea pipelines. The TEG was generating energy from the temperature difference between the pipe and the water. The collected temperature and motion data of the pipe was relayed using optical wireless communication. Usually thermoelectric generators are considered by designers when a significant thermal energy source is available (either cold or hot), providing tens of degree C difference with respect to the environment, however, authors of [27] demonstrated the possibility to recharge a super-capacitor with a thermal gradient as low as 0.6 °C, paving the way to new application scenarios.

## 3. Method and Approach

The purpose of our work is building a self-powered smart bolt that senses its own temperature, tension and other physical parameters and transmits the sensed data to a central controller over sub-GHz radio. Our approach is first modularizing the system into three units namely the power generation unit i.e., TEG, the power conditioning unit i.e., DC-DC boost converter and storage capacitor and finally the core system which is composed of a CC1310 microcontroller and sensors. The TEGs used in this work are Peltier modules or thermoelectric coolers (TECs).

The primary objective of this work was not achieving accuracy and higher performance in the sensing side but rather the focus was in making the system energy neutral using TEG, considering that usually systems, typically employed for structural monitoring, exploit other environmental energy sources [28]. This requires conducting an accurate power budget analysis of each sub-system under different schemes. Accordingly, the TEGs were characterized to record the amount of power they can generate under different conditions. The DC-DC converters were also characterized for their efficiency and finally the power consumption of the prototype system was analyzed under different conditions.

### 3.1. Characterization of TEGs

The power density and the power density per square temperature gradient (power factor) are the two important parameters that uniquely identify the performance of a TEG. The design of the self-powered bolt started from characterization of three commercial thermoelectric generators from Digi-Key distributor namely 926-1216-ND (TEG1), 926-1192-ND (TEG2), and 926-1225-ND (TEG3). Table 1 contains the summary of the specification of the TEGs. The characterization included measuring (i) the TEG output power at different values of load resistors and at a constant temperature gradient, ΔT, between the faces of the TEG and (ii) the TEG output power at different ΔT values at a matched load.

The first test was done by keeping ΔT constant while varying the load resistance and recording the current and load voltage values to find the maximum power. This test was repeated for several ΔT values with a waiting time of at least 30 min to let the temperature reach a steady state and a uniform distribution on the heating plate. We used a series of four 1 Ω, 25 W resistors supplied with a 12 V DC (max 200 W) source and an analog potentiometer to regulate the heat, mounted on the bottom of an aluminum plate to realize a uniform heating plate, while a 40 × 40 × 15 mm^3^ aluminum heat-sink was placed on the cold TEGs’ side. Finally, three NTC thermistors were used to collect hot-side, cold-side and environmental temperatures during tests. The selected range of the temperature gradients provides a better understanding of the TEGs properties. The values of ΔT ranged from 5 °C to 40 °C. During these tests, the temperature of the hot side did not exceed 80 °C. The resistor values used for this test ranged from 0.8 Ω to 10 kΩ. Then the gathered data was processed using MATLAB to determine maximum power point of the TEG for each ΔT. The second test was done by recording the output power of the TEG by using a matched resistor load and varying ΔT values.

**Figure 2:**
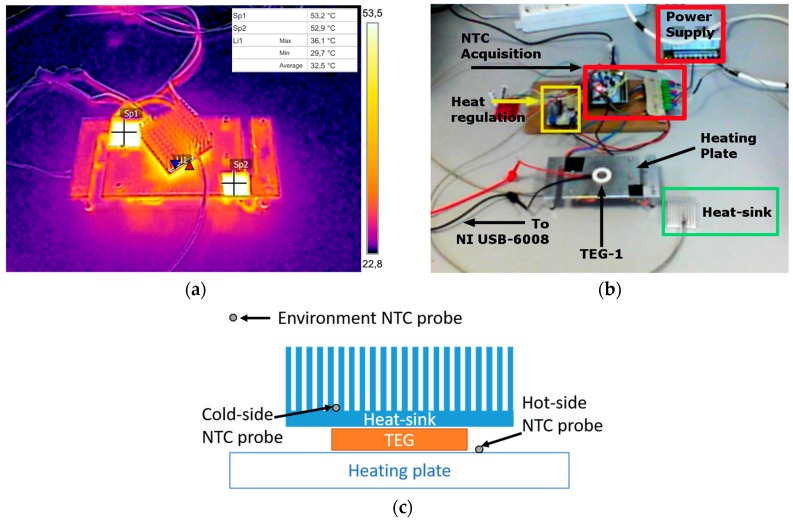
Measurement setup, thermal image of the experimental setup working at around 50 °C, bright spots are the reference points of known reflectance for thermal imager (**a**), block description of the sub-systems (**b**), schematic layout of Negative Temperature Coefficient (NTC) thermistor probe positioning (**c**).

**Figure 3:**
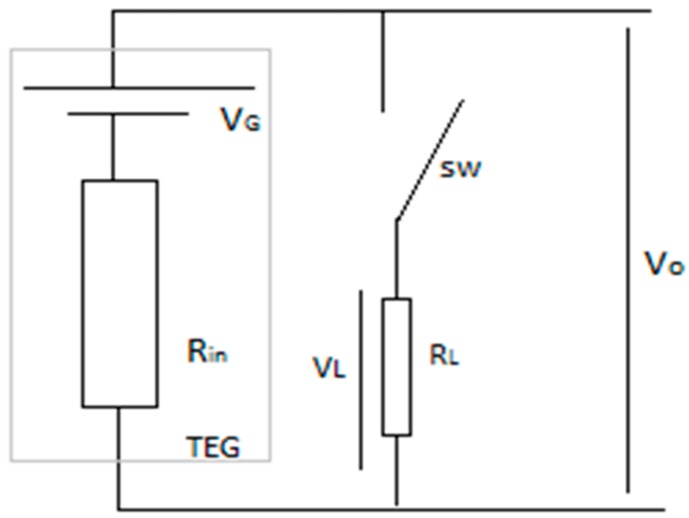
Circuit used for characterization.

The current in the circuit and the voltage at the load were continuously recorded using NI LabVIEW USB-6008 acquisition tool from National Instruments [29]. Additionally, a µCurrent [30] amplifier has been used to adapt the very small current generated by TEGs with the input range of the acquisition tool. The acquisition board was sampling data at 100 Hz and was feeding it to a LabVIEW project on a PC that was sorting and storing the measured values. The temperature at the faces of the TEGs were measured using a NTCLE100E3 thermistors whose R25 = 10 kΩ. Pictures of the measurement setup and related schematics are shown in Figure 2, while the circuit used for the acquisition and characterization of TEGs is depicted on Figure 3.

When current starts to flow in the measurement circuit the temperature gradient, ΔT, between the faces of the TEG starts to fluctuate due to the Peltier effect [15]. The fluctuation of ΔT in turn causes the fluctuation of the Seebeck coefficient. Therefore, an external thermostating circuit should be used to keep the temperature at a set point while conducting measurement. In [15], the thermostating was implemented using two extra TEGs, one as a heater and the other as a cooler, controlled in a feedback loop according to the temperatures of the faces of the main TEG. While providing a reliable measurement data, this solution has the disadvantage of adding complexity to the simple circuit used for acquisition. In our work a different approach was followed to overcome this problem. First the fluctuation of ΔT in relation to the current was observed from repetitive measurements using the circuit shown in Figure 3 which is a circuit without thermostating. This led to identification of a pattern as to how ΔT was varying with the current. Then a software filter was implemented in Matlab to correct the fluctuation and gain correct output power values despite the fluctuation. Hence, instead of building a complex thermostating circuit the simple characterization circuit of Figure 3 was kept as is.

A heat sink was used for cooling and to provide a mechanical load on the TEG. Mechanical pressure on the TEG helps to reduce thermal contact resistance and creates a uniform temperature distribution on the surface of the TEG.

### 3.2. Characterization of DC-DC Converters

Two DC-DC boost converters were selected for the characterization, namely the LTC3108 from linear technologies [31] and the Nextreme WPG-1 from formerly Nextreme now Laird Technologies [32]. The former one is an ultra-low voltage step-up integrated circuit that can work with input voltages as low as 20 mV, has selectable output voltage level with maximum 10 mA current and, finally it does not integrate maximum power point tracking (MPPT) features. Also, the latter one has no MPPT since it is based on the Texas Instruments BQ25504 boost converter [33], with a minimum input of 80 mV and 1 mW maximum output power with selectable voltage range. A super capacitor was connected to the output of both boost converters to store energy coming from the TEG when the load was in low power mode. The converters were characterized for their efficiency and charging profile of the output 250 mF supercapacitor. The converters were supplied once from the TEG and at another time from a DC source (Keysight E3648A [34], while in both cases their input and output powers were recorded simultaneously. The efficiency of DC-DC converters varies in relation to the input power therefore supplying the converter from the DC source was necessary to assess the efficiency of the converter at higher input power.

### 3.3. Measuring the Power Requirements of the System

The smart bolt consists of CC1310 microcontroller [35] interfaced with a temperature sensor and a tension sensor and it was powered from a TEG. The system was running a TI-RTOS based firmware and the MCU was programmed and debugged using SmartRF06 [36] evaluation module. The power consumption of the MCU was measured by varying the transmission power, packet size, duty cycle and type of modulation.

## 4. Results and Discussion

### 4.1. Results from the Characterization of TEGs

The characterization of the thermoelectric modules was conducted to verify the data reported by the manufacturer before employing the TEGs for use and to obtain more information about the power density and the power factor, parameters that were not reported on the technical document provided by the manufacturer. The results from the experiments are summarized on Table 2. The table contains results obtained from the TEGs in our experiments (the first three entries) and for comparison it also includes results reported on other TEG modules (the last four entries in Table 2) by other authors. The characterization result of TEG4 (PE127-14-15) and TEG5 (Thermolife 009r) are as reported in [15] and the results for TEG6 (Peltier module 12706AC) and TEG7 (TEC-12710) are reported in [37] respectively. Due to their small surface area, TEG2 and TEG3 have a very low PmaxΔTmax2 but their power factor exceeds TEG1’s power factor in orders of magnitude. A similar comparison of power factors of the TEGs on Table 2 reveals that TEG2 and TEG3 have the best performance. Based on the results, the TEGs that were used in this work had a better performance than the TEGs they were compared with.

**Figure 4:**
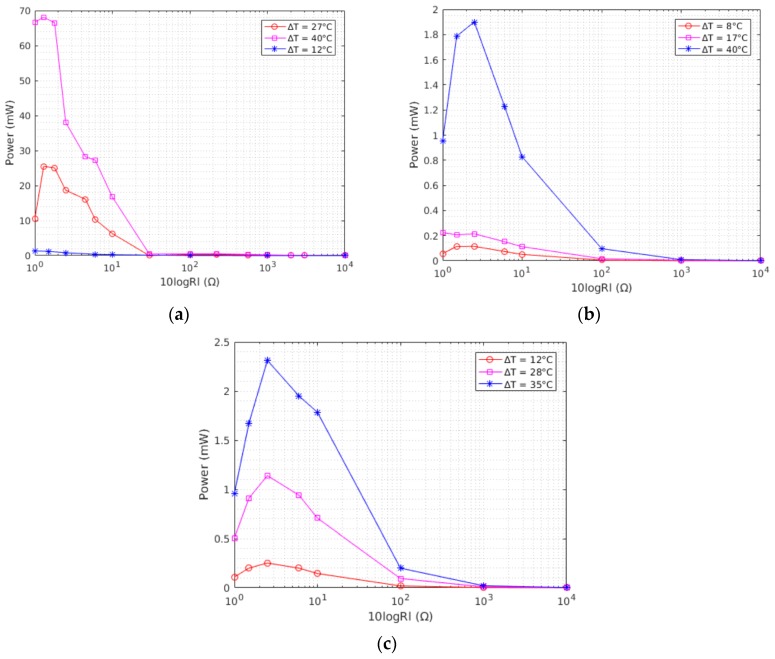

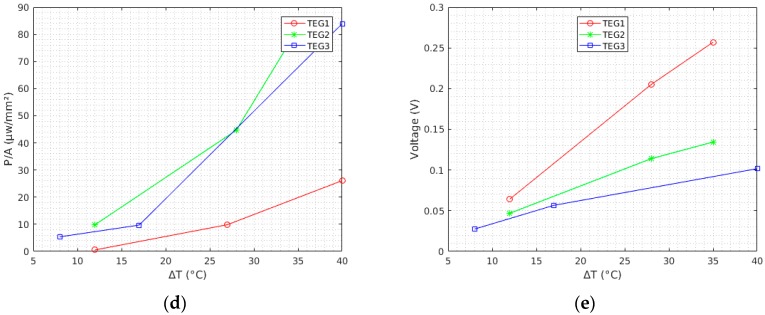
Power vs. load (log scale) for (**a**) TEG1; (**b**) TEG2; and (**c**) TEG3; (**d**) Power vs. ΔT at matched load for the three TEGs; (**e**) Open circuit voltage vs. ΔT for the three TEGs.

Figure 4a depicts the variation of the output power of TEG1 with the load resistance under different temperature gradients (accordingly Figure 4b,c report results for the other two devices). As shown in the graph, as the resistance of the load increases the output power is severely reduced. The TEGs used in this work are Peltier modules which are designed with a low internal resistance to increase the current flow and therefore to absorb more heat when a constant voltage is applied. There is also a discrepancy between the manufacturer reported internal resistance values as summarized in Table 1 and the internal resistance values measured in the lab (Table 2). This is because selected commercial TEGs are designed as system for thermal management, and, specifically, to reach very low cool down temperatures. Their design has not been optimized for harvesting applications and this different kind of utilization, namely extracting power from thermal gradients instead of pumping energy to generate it, is the main cause of this discrepancy. Moreover, the characterization has been performed using discrete resistors at different temperature gradients thus values reported in Table 2 represent a numerical estimation obtained fitting the curves presented in the following.

While having information about the maximum power from a TEG is very important to plan the power budget of an application, it is also more important to have a measure of the power factor and power density to correctly understand the capabilities of a TEG and making the right decision in the design choice stage. The power density (power per unit area) vs. ΔT at a matched load for each module is shown in Figure 4d. Even though TEG1 generates higher power compared to the other TEGs, due to its larger surface area, it has a lower power density compared to the other two TEGs.

The open circuit voltage and ΔT also have a linear relationship and this is clearly depicted on Figure 4e. TEG1 has the highest open circuit voltage for a temperature gradient compared to the two TEGs. The knowledge of the open circuit voltage under different temperature gradients is useful to correctly choose or size the input stage of any energy harvesting circuitry (or to tune the maximum power point tracking stage in the case it is available, not considered in this work).

### 4.2. Results from the Characterization of DC-DC Converters

**Figure 5:**
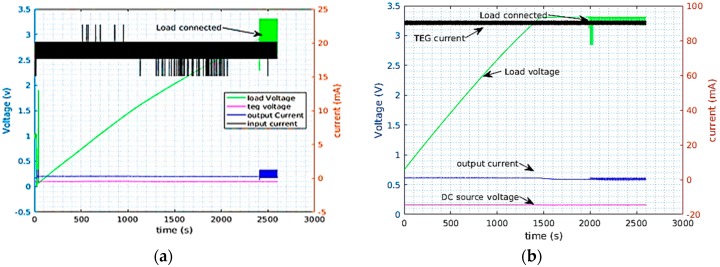
LTC3108 supplied from TEG with 15 °C thermal gradient (**a**) and a DC source (**b**).

**Figure 6:**
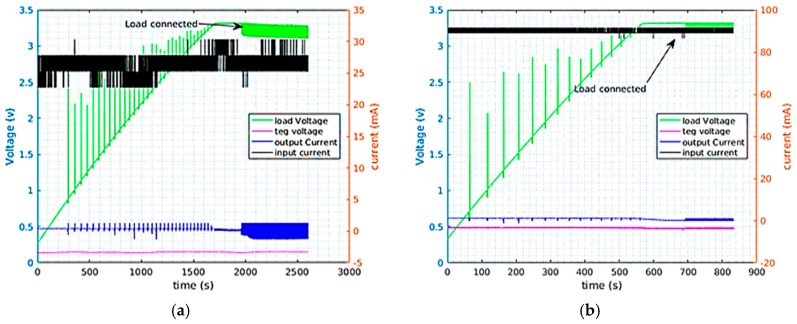
Nextreme supplied from TEG with 15 °C thermal gradient (**a**) and a DC source (**b**).

In low energy harvesting, the power scavenged from TEGs is used to supply electronic devices continuously or with some duty cycle. TEGs provide adequate power but with very low nominal voltages. For example, TEG 1 was generating 18 mW at a temperature gradient of 20 °C at 140 mV. This power is considerably large but the low voltage from the TEG is not enough to start almost any kind of electronic device. Hence, to use the TEG power, the TEG voltage needs to be boosted using DC-DC boost converters. A prototype load which replicated the behavior of the MCU with a radio was used for the characterization of the converters. This load was simulating a radio communication with a duty cycle of 0.5% and it was running with a period of 3 s. It was consuming 42.15 mW in the active mode and 360 µW in low power mode. Figure 5 and Figure 6 depict the recorded measurements when the LTC3108 and Nextreme modules were being supplied by a TEG and DC source respectively. On Figure 5a for example, the TEG was depicted as charging the supercapacitor connected to the LTC3108 boost converter, while Figure 5b shows the case when DC supply is employed. The load was connected to the supercapacitor only after the supercap voltage reached 3.3 V. In this test the TEG current, TEG voltage, the current to the supercap and the supercap voltage were simultaneously recorded. Figure 6 depicts similar measurements for the Nextreme converter. The result of the characterization of the converters is summarized in Table 3 and Table 4, while the thermal gradient used to conduct tests with TEGs was kept around ~15 °C.

As the input power increases the efficiency of both converters decrease significantly. From the results of the efficiency the LTC3108 outperformed the Nextreme-WPG-1 module in both cases. However, when supplied from the TEG it took the Nextreme module about 25 min to charge the supercap to 3 V while it took the LTC3108 close to 40 min to do the same. Even though the LTC3108 had a higher efficiency (almost double) it was receiving less input power from the TEG (because of the different working point) and therefore it took longer time to charge.

Thus, in conclusion, the optimal choice depends on the application, one could prefer higher efficiency (longer recharge time) in case of applications with very low duty-cycle, or a faster recharge (with higher wastes) when the startup time is crucial. In this work, we opted for the most efficient LTC3108 solution.

**Figure 7:**
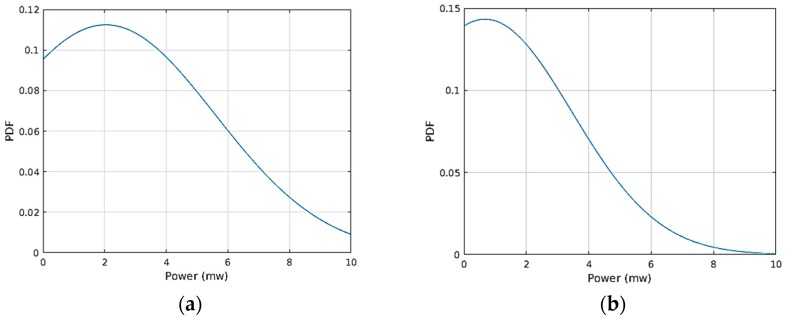
Probability distribution of output power in the Nextreme WPG-1 module when supplied from (**a**) DC source and (**b**) TEG.

The supercap charging power was not constant. To calculate the efficiency of the converter the average output power was used. The average power was calculated first by forming a set consisting of the rate of change of energy at the capacitor every 330 ms into a set. The set was then observed to have a normal distribution as depicted in Figure 7. The mean of this distribution was then taken as the average power and it was used to estimate the efficiency. Figure 7a,b depict the normal distribution of the average output power when the Nextreme module was charging the supercap when the module was supplied from the DC source and the TEG, respectively. The mean output powers were around 2.6 mW for the DC source and 0.9 mW for the TEG. Similarly this procedure was repeated for the LTC3108 IC, to extract characteristics used in the following, not shown for the sake of summary.

### 4.3. Results from the Characterization of the System

The smart bolt system consists of a CC1310 low power wireless MCUs from Texas instruments and a TC3108 DC-DC boost converter. For this experiment, the system was powered using TEG1. Despite their higher power factor, TEG2 or TEG3 are too small to provide enough total power for the final application; however, given their small size a number of these TEGs can be combined in series to suit the power budget of the application and the volume of the final system. Although TEG1 has a relatively larger footprint (1.5 ${\mathrm{cm}}^{2}$) compared to the other two TEGs, it can still easily fit on the top of most critical fasteners. The CC1310 has an ARM cortex M3 CPU running with a 48 MHz clock. For the prototype system, temperature readings from the internal temperature sensor of the MCU were used as transmitted data. The MCU also has an ARM M0 core that runs the radio firmware. The radio communicates with the software running on the main CPU using a shared memory interface. For this test, another CC1310 module was used as a receiver to record correct reception of transmitted packets.

The energy consumed by the microcontroller was then measured (i) by varying the transmission power and the type of modulation while keeping the packet size and transmission period constant; (ii) by varying the packet size and transmission period under a fixed transmission power and modulation; and (iii) by varying the transmission power, type of modulation and packet size under a fixed transmission period. In all cases, the transmission frequency was 868 MHz. All the unused GPIOs were connected to the internal pull resistor to protect current leakage. The device power management is handled by TI-RTOS. With minimal or no configuration in the application, the RTOS by default invokes a target specific power policy during the idle time of the application. In this test, since the application, running on the Cortex M3 was utilizing only the radio and no other peripherals both CPUs switch into standby mode immediately after transmission. The wakeup time of the radio was observed to be 0.9 ms.

**Figure 8:**
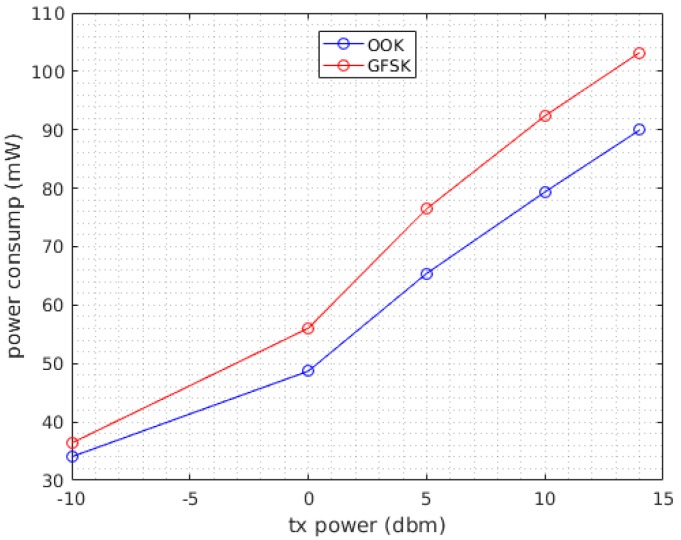
Transmitter power consumption under (**blue**) On-Off keying (OOK) modulation and (**red**) Gaussian frequency-shift keying (GFSK) modulation.

**Figure 9:**
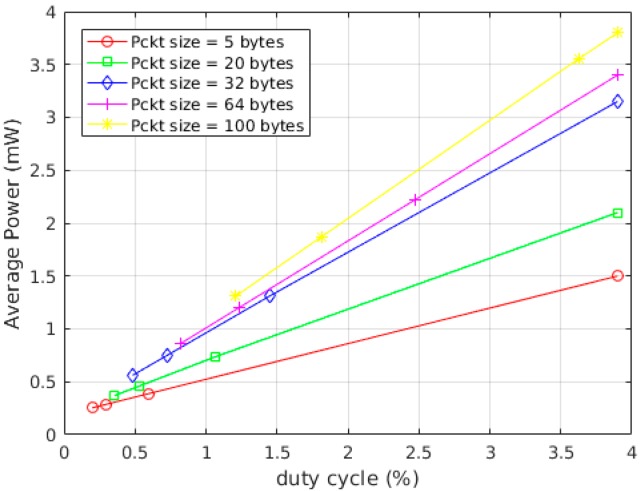
Correlation between power consumption, packet size, and duty cycle at 14 dbm transmission.

In case (i) the power consumption of the system during transmission was measured by varying the transmission power values from −10 dbm to 14 dbm in Gaussian frequency-shift keying (GFSK) and On-Off keying (OOK) modulations. The packet size was 32 bytes and the transmission was repeated every second for both modulations. For a fixed transmission power, the amount of current drawn by the system remains constant even when varying the packet size and duty cycle. In fact, the variation of these parameters only changes the duration the system stays in the active mode and hence only changes the energy consumption. The result of this test is represented in Figure 8. As depicted in the plot, the usage of GFSK modulation resulted in a slightly higher power consumption by the system. In case (ii) the power consumption of the system was measured by varying the packet size and the transmission period, meanwhile keeping the transmission power constant at 14 dbm. The radio was transmitting in GFSK modulation while the packet sizes that were tested were 5, 20, and 64 bytes with transmission duration of 2.96 ms, 5.37 ms, and 12.39 ms respectively. The transmission was repeated with periods of 500 ms, 1 s, and 1.5 s resulting in duty cycles of [0.592%, 1.074%, 2.47%], [0.296%, 0.537%, 1.23%], and [0.197, 0.358%, 0.826%], respectively, for each packet size. Figure 9 shows the relationship between duty cycles and the average power consumption over different packet sizes.

Based on the results of the first test, the system was consuming 102 mW when transmitting at 14 dbm and 240 μW in the low power mode. The power consumption reported in this test is the average power consumption in the active and low power modes which is calculated by dividing the total energy in the active mode and low power mode by a single transmission period. As inferred from Figure 9, the average power is directly related to the packet size and the duty cycle. As the size of packet increases the current drawn by the MCU and hence the energy consumption increases considerably.

**Figure 10:**
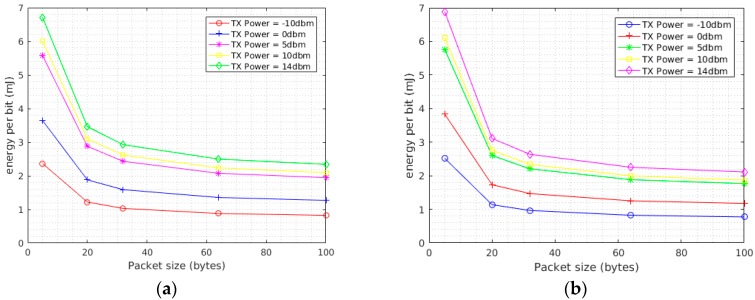
Transmitter power consumption under different packet sizes and transmission power in (**a**) GFSK modulation (**b**) OOK modulation.

For case (iii) the power consumption of the system was measured over packets of sizes of 5, 20, 32, 64, and 100 bytes and set of transmission power levels ranging from −10 dbm to 14 dbm. The purpose of this test was further quantifying the performance of the system by measuring the energy per bit under a set of changing parameters. The energy per bit values obtained from this test will ultimately be used by the microcontroller for a choice of transmission parameters based on the available energy at the supercapacitor and sensed channel condition. The transmission period was one second. The measured power consumption was then used to calculate the energy per bit. The measurement was done for both the GFSK and OOK modulations. Also, in this case the average energy from the average power was used to calculate the energy per bit. As seen on Figure 10, GFSK has a lower energy per bit at lower packet sizes and the reverse is true as the packet size starts to increase. For example, for a 5 byte packet the transmission of a single bit in GFSK at 14 dbm has an energy cost of 6.9 mJ while the same transmission in OOK costs about 7.1 mJ. At a packet size of 32 bytes, the energy per bit at 14 dbm is about 3.9 mJ in GFSK while it is 3.6 mJ for OOK. The same pattern repeats for different transmission power values and packet sizes. From the results obtained at lower packet sizes, the energy per bit is slightly lower when using GFSK and in reverse using OOK results in a lower energy per bit in larger packets. The average power in this case is calculated by first computing the total energy consumed by the module in each period and dividing this energy by the period.

**Figure 11:**
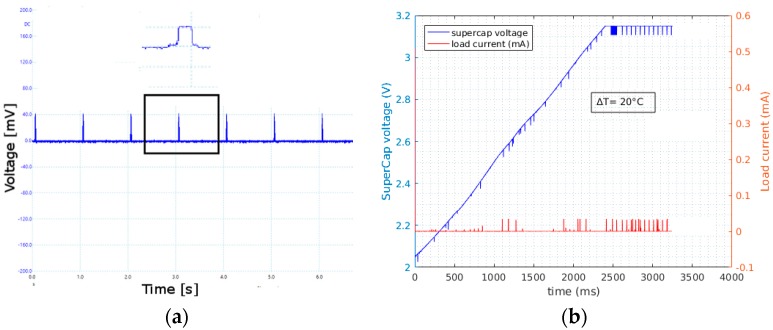
(**a**) Current consumption of the duty-cycled system with detailed view in the inset, here 1 mV equals 1 mA thanks to the µCurrent amplifier. (**b**) Voltage and Current at the supercap when the load is connected.

The results of the prototype system are depicted on Figure 11. Figure 11a represents the power consumption of the system during transmission and in low power modes when the system was transmitting with a period of 1 s, collected using Picoscope USB oscilloscope and the µCurrent amplifier, where 1 mV corresponds to 1 mA. Figure 11b presents an excerpt of the current drawn by the system along with the voltage at the supercapacitor. The system was powered from TEG1 with a temperature gradient of 20 °C with respect to the environment. The system was sending packets of 32 bytes every second at 14 dbm. The test was done to measure the performance of the final system and assert the results obtained so far. The voltage of the supercap started from 0 V and the load was connected from the beginning of the test. The system started operation after the voltage from the DC-DC converter rose above 2.5 V (around second 1.1 in Figure 11b) and this took a duration of 16 min. The charging of the supercap reached 3.1 V in 45 min and the system continued to function in a self-sustainable manner.

After the power generation and consumption analysis of the different modules and components of the system, and the radio performance, we focused on the study of the expected performance of the whole smart bolt. The performance of the system evaluated by setting the transmission power to 14 dbm and using GFSK modulation. The packet size was also set to 32 bytes and it had a transmission time of 7.26 ms. Then the maximum duty cycle at which the system remains energy neutral under different temperature gradients is computed by accounting the power generated by the three TEGs at different temperature gradients and the energy consumed by the system while transmitting. Table 5 is a summary of the results. The values in Table 5 are calculated using Equation (3) which was derived from Equations (1) and (2).

(1) $$E_{tot}=E_{active}+E_{sleep}=P_{in}T=P_{tx}t_{tx}+P_{sleep}\left(T-t_{tx}\right)$$

(2) $$T=\frac{P_{tx}t_{tx}-P_{sleep}t_{tx}}{P_{in}-P_{sleep}}$$

(3) $$\mathrm{Duty}-\mathrm{Cycle}=\frac{t_{tx}}{T}$$

In Equation (1), $E_{tot}$, $E_{active},\;\mathrm{and}\;E_{sleep}$ are the total energy, energy consumed while the system is active, and in low power mode, respectively. $t_{tx}$ is the transmission time and *T* is the sum of transmission and sleep times. $P_{in}$ is the power at the load and it is obtained by multiplying the power generated by TEG1 at different temperature gradients (in Figure 4a) by the efficiency of the DC-DC converter. $P_{tx}$ is the power consumption of the system during transmission of packets and it was determined in the characterization of the power consumption of the load (Figure 6). $P_{sleep}$ is the power consumed by the load in low power mode and it was determined to be 240 μW. The maximum duty cycle for each power level is then calculated by determining the minimum amount of time the system must remain in low power mode to store enough energy for the next transmission. For instance, to stream a packet of 32 bytes (7.26 ms transmission time) at a temperature gradient of 10 °C, the system must transmit with a minimum period of 468 ms (1.55% duty cycle). One interesting result from this summary was understanding that TEG2 and TEG3 are not able to support the application at a temperature gradient below 14 °C.

### 4.4. Discussion

The results of all the experiments done in this work are not used only at the design stage for choosing the right type of TEG or DC-DC converter but are also used by the firmware to adaptively decide radio transmission parameters at run-time. The firmware that is running on the cortex M3 receives a temperature reading and the energy level of the supercap. The temperature reading which is going to be transmitted to a central controller is also used by the application to estimate the amount of power that is being generated by TEG using characterization information in Section 3.1. The application then measures the amount of energy stored in the supercapacitor.

**Figure 12:**
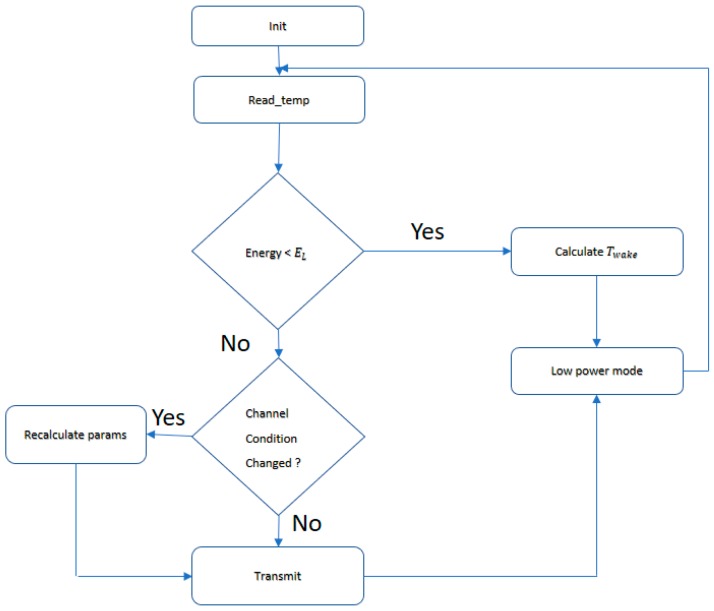
Flowchart of the adaptive parameter transmission adjustment.

Based on these two values (TEG power and energy in the supercap) and assessment of the channel from previous transmissions the application makes a revision on the transmission power and the duty cycle values. If the energy in the supercap is below a threshold, $E_{L}$, the application takes the system into a low power mode after scheduling the next wakeup to $T_{wake}$. $T_{wake}$ is the time the TEG takes to charge the supercap to $E_{H}$ and it is easy to calculate as the application is already aware of the amount of power the TEG is generating and the charging profile of the supercap by the DC-DC converter from Section 3.2. The application flow is depicted in Figure 12.

In the current stage of deployment, only the internal temperature of the MCU is available, while the interfacing of external NTC probes and pressure/strain sensors is under development. This information is used as the estimation of the hot-side temperature since the very low power consumption of the MCU and the very low duty cycle (active time is few tens of ms for the whole task) result in negligible heat dissipation by the electronics.

## 5. Conclusions

In this work, the design of an energy neutral wireless bolt was investigated. The system transmits temperature of the bolt to a central controller in the sub-GHz band and it consisted of TEG, a CC1310 MCU from TI and a LTC3108 DC-DC converter. There Peltier modules, which were used as TEGs were characterized to evaluate their performance. The characterization of two DC-DC converters was also done to determine their efficiency. Finally, the power consumption of the system under different transmission parameters was recorded and this result was then used to design an algorithm which determines the duty cycle and transmission parameters of the system based on the available energy and channel conditions. The firmware of the system was TI-RTOS based and it was possible to stream temperature data every 488 ms (1.55% duty-cycle) with a ∆T as low as 10 °C.

## Figures and Tables

**Table 1:** Summary of Specifications of thermoelectric generators (TEG) from datasheet.

Label	Model	L [mm]	H [mm]	Rin [Ω]	ΔTmax [K]	A [$mm^{2}$]
TEG1	926-1216-ND	26	14	0.25 @ 25 °C hot side T	67	1507
TEG2	926-1192-ND	5	3.4	1.04 @ 25 °C hot side T	67	17
TEG3	926-1225-ND	3.9	3	dnp	92	15.21

**Table 2:** Summary of experimental results and comparisons with reference works in literature.

Label	Rin [Ω]	PmaxΔTmax2 [$\mu w/K^{2}]$	PF $\left[\mu w/mm^{2}k^{2}\right]$
TEG1	1.4	40.625	0.0271
TEG2	2.3	6.94	0.404
TEG3	2.1	6.04	0.397
TEG4	2.23	224	0.14
TEG5	250 K	1	0.015
TEG6	1.9	5.31	0.0033
TEG7	1.08	0.25	0.156

**Table 3:** LTC3108 Characterization Result.

Supply	Vin [mV]	Iout [μA]	Pin [mW]	Pout [mW]	η (*%*)
TEG	78	250	1.69	0.83	49.1
DC	477	1000	42	3.16	13.29

**Table 4:** Nextreme Characterization Result.

Supply	Vin [mV]	Iout [μA]	Pin [mW]	Pout [mW]	Η (%)
TEG	136	380	3.4	0.95	27.1
DC	477	850	43.5	2.6	5.9

**Table 5:** Estimation of max duty cycle based on input power.

Label	Experimental Result					
	ΔT=10		ΔT=20		ΔT=30	
TEG1	$P_{in}$ (mW)	1.8	$P_{in}$ (mW)	16.4	$P_{in}$ (mW)	44.5
	%d	1.55	%d	18.9	%d	77
TEG2	$P_{in}$ (mW)	0.18	$P_{in}$ (mW)	0.87	$P_{in}$ (mW)	2.0
	%d	-	%d	0.62	%d	1.76
TEG3	$P_{in}$ (mW)	0.15	$P_{in}$ (mW)	0.45	$P_{in}$ (mW)	1.4
	%d	-	%d	0.2	%d	1.15
